# Advancing chemokine research: the molecular function of CXCL8

**DOI:** 10.1172/JCI180984

**Published:** 2024-05-15

**Authors:** Yiran Hou, Anna Huttenlocher

**Affiliations:** 1 Department of Medical Microbiology and Immunology and; 2 Department of Pediatrics, University of Wisconsin School of Medicine and Public Health, Madison, Wisconsin, USA.

## Abstract

CXCL8 and other chemokines have been implicated in tissue inflammation and are attractive candidates for therapeutic targeting to treat human disease.

Neutrophils are critical for host defense against infection. Central to neutrophil function is the infiltration into damaged tissues mediated by a multistep process that requires the coordination between both immune and nonimmune cells. Among factors that facilitate neutrophil recruitment is the chemokine CXCL8, one of the most well-studied chemokines. In the JCI in 1989, Baggiolini and colleagues described CXCL8 (also known as NAP-1 and IL-8) as a “novel cytokine that activates neutrophils” (1). Identification of CXCL8 followed the discoveries of other neutrophil chemotactic factors, including the bacterial peptide fMLP, anaphylotoxin C5a, and lipid mediator LTB4. However, progress in identifying CXCL8, its corresponding receptors, and downstream signaling transformed the field of chemokine biology. Many past Reviews have covered the discoveries related to CXCL8 and its receptors (2). Here, we discuss the role of models and tools in advancing chemokine research and recent advances in understanding new functions for CXCL8 and its receptors.

## Early discovery of chemokines

Recruitment of neutrophils is required for host defense responses, but it also contributes to inflammation and tissue damage. By the 1980s, specific modulators controlling neutrophil recruitment were identified using isolated leukocytes and human skin disease models (3). External modulators like LPS were found to indirectly induce neutrophil chemotaxis through the generation of “host defense cytokines” or chemokines (4). Using crude purification, the cytokine IL-1 from LPS-stimulated mononuclear cells was identified as the internal factor that regulates neutrophil recruitment. However, further biochemical separation identified the actual functional peptide, now known as CXCL8 (4). In 1987, three teams independently purified CXCL8 using similar approaches, separating CXCL8 from IL-1 by its net charge, size, and hydrophobicity (1). They further tested its chemotactic ability, demonstrating that CXCL8 functions as a potent neutrophil chemoattractant ex vivo.

Since the identification of CXCL8, more research into the mechanism of its attractive effects on neutrophils has followed. From the receiver side, biochemical and biophysical measurements revealed that neutrophils show an increase in cytosolic Ca2+, shape changes, superoxide generation, and granule exocytosis upon CXCL8 treatment (5). Identifying the receptor for CXCL8 proved to be a challenge. Back in 1990, no prior knowledge existed for what a chemokine receptor might look like. Therefore, researchers took an unbiased approach, where they first screened cDNA libraries generated from human neutrophils, searching for genes whose products bound to CXCL8 and induced intracellular Ca2+ increases following expression in cells (6). This led to the discovery of CXCR1, a high-affinity receptor for CXCL8, and the finding that chemokine receptors belong to the family of G protein–coupled receptors. With one sequence in hand, further screening was expedited by homology hybridization, uncovering CXCR2, which also mediates CXCL8 signaling.

In 1989, only two years after its initial purification, the structure of CXCL8 was determined by nuclear magnetic resonance and later resolved with X-ray crystallography (7). Two key points were inferred from the structure: CXCL8 forms hydrogen bond-stabilized dimers and its residues 4–9 and 31–38 could be important for receptor binding. The importance of residues 4–6 was soon confirmed by experimenting with synthetic CXCL8 truncated in different regions, identifying the ELR motif necessary for receptor binding (5). These findings provided a solid foundation for understanding the molecular function of CXCL8; however, these advances alone were insufficient to understand the physiologic role for CXCL8 in vivo.

## Roles for CXCL8 and its receptors

**Figure 1:**
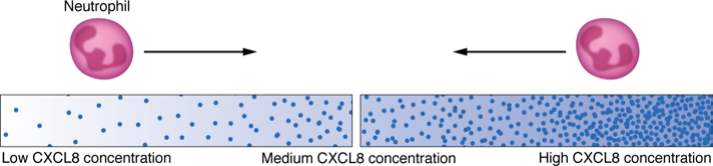
CXCL8 concentration gradient determines neutrophil migration pattern. Neutrophils migrate toward regions with higher concentrations of CXCL8 when the CXCL8 gradient ranges from low to medium concentrations. This directionality can reverse when there are high concentrations of CXCL8.

Substantial in vitro data characterized how CXCL8 regulates neutrophil motility. CXCL8 can induce neutrophil chemokinetic movement in addition to providing a chemotactic or directional cue. Real-time imaging of neutrophil motility in vitro using different matrix conditions and microfluidic devices has provided even more information about how CXCL8 or other chemokines regulate neutrophil motile behaviors. While neutrophils migrate toward increasing concentrations of CXCL8, high concentrations of CXCL8 can repel neutrophils and induce their movement away from a source of chemoattractant (Figure 1) (8, 9). These findings suggested that CXCL8 not only serves as a chemoattractant, but can also induce movement of neutrophils away from a source of chemoattractant in vitro.

The challenge to understanding CXCL8 function in vivo has been finding the right model system that expresses endogenous CXCL8. Early studies in mice showed a conserved role for human CXCL8 in neutrophil recruitment and the requirement of CXCR2 for neutrophil infiltration (1). However, the gap in understanding the role of endogenous CXCL8 remained because mice do not have a homolog of human CXCL8. The tides turned in the early 2000s, when zebrafish were found to express homologs of CXCL8, CXCR1, and CXCR2 (10). Zebrafish provide a robust alternative model, because the innate immune system is highly conserved and the optical transparency enables imaging of neutrophil migratory behavior in interstitial tissues. Indeed, live imaging of zebrafish neutrophils identified the process of neutrophil reverse migration away from wounds to resolve local tissue inflammation (10). Knockout of cxcl8 and cxcr2 results in a defect in neutrophil reverse migration and impaired resolution of neutrophils at wounds (11). The receptors CXCR1 and CXCR2 have differences in receptor trafficking that enable the initial recruitment by CXCL8 and the subsequent resolution of neutrophils at wounds. In the wound, activated CXCR1 is rapidly internalized, whereas CXCR2 persists on the plasma membrane and this mediates sustained signaling, which supports chemokinesis and the subsequent neutrophil reverse migration (12). Further studies in zebrafish have shown that CXCL8 establishes tissue-bound gradients in vivo by binding to proteoglycans in the extracellular matrix, providing a more stable tissue cue that also guides neutrophil behavior (13). Therefore, CXCL8 can be presented as both a soluble and insoluble directional cue that influences neutrophil motile behavior and positioning in tissues.

## Looking forward in disease

CXCL8 and other chemokines have been implicated in tissue inflammation and have been attractive candidates for therapeutic targeting to treat human disease. However, this approach has not been highly successful, in part, because, like CXCL8, chemokines can have both anti- and proinflammatory effects. Going forward, it is critical to have strong human in vitro models to understand the complex function of chemokines. The use of neutrophils derived from human induced pluripotent stem cells that can be genetically modified to increase understanding of neutrophil motility signaling and interactions with endothelium in organotypic models has recently been demonstrated (14). Technological advances are expanding the range of what we can do. For example, omic approaches that can be used to understand how CXCL8 and its receptors are altered in disease states are now accessible (15). For chemokine research in particular, understanding interactions between receptor and ligands within different tissue contexts in humans is key. With a more holistic view of chemokine regulation in patient contexts, we will gain insights into the pathways that are used in specific subpopulations of cells that can be fine-tuned to achieve immunoactivation or immunosuppression as needed.

## Closing remarks

In fewer than 40 years, chemokine research, chemokine research has made rapid progress in understanding how these small peptides regulate tissue inflammation. We now know how signals transmit from chemokine producers to its receivers and how chemokines contribute to multiple cell functions. Although these findings have offered abundant therapeutic targets for drug development, the bench-to-bedside process remains challenging because of the molecular and functional redundancy in chemokine signaling and difficulty in understanding their spatial-temporal regulation in diseased tissues (16). These challenges can be overcome by advances in structural biology to capture chemokine-receptor conformation dynamics and finer resolution of endophenotype readouts. With new models and technologies, we anticipate the development of novel treatment strategies that are more specific and provide promise for human disease treatment.

## Version 1 05/15/2024

Electronic publication
